# Increase in isolation of extended spectrum beta lactamase producing multidrug resistant non typhoidal Salmonellae in Pakistan

**DOI:** 10.1186/1471-2334-10-101

**Published:** 2010-04-22

**Authors:** Kauser Jabeen, Afia Zafar, Seema Irfan, Erum Khan, Vikram Mehraj, Rumina Hasan

**Affiliations:** 1 Department of Pathology and Microbiology, Aga Khan University, Karachi, Pakistan

## Abstract

**Background:**

Increasing resistance to quinolones and ceftriaxone in non typhoidal Salmonellae is a global concern. Resistance to quinolone and 3^rd ^generation cephalosporin amongst non typhoidal Salmonellae (NTS) from Pakistan has been reported in this study.

**Methods:**

Retrospective analysis of laboratory data was conducted (1990-2006). NTS were isolated and identified from clinical samples using standard microbiological techniques. Antimicrobial susceptibility testing was performed by Kirby Bauer. Extended spectrum beta lactamase production (ESBL) was detected using combined disc method. Ciprofloxacin sensitivity was detected by nalidixic acid screening method. Minimum inhibitory concentration (MIC) of ciprofloxacin was determined by agar dilution method. Statistical analysis was performed using SPSS version 13.

**Results:**

Analysis of 1967 NTS isolates showed a significant increase in ciprofloxacin resistance from 23% in 2002 to 50.5% in 2006, with increased mean MIC values from 0.6 to 1.3 ug/mL. Ceftriaxone resistant NTS also increased and ESBL production was seen in 98.7% isolates. These isolates exhibited high resistance against amoxicillin clavulanic acid (57%), gentamicin (69%), amikacin (44%) and piperacillin tazobactam (30%). No resistance to carbapenem was seen. Ceftriaxone resistance was significantly higher in children <1 year, in invasive isolates and in Salmonella Typhimurium.

**Conclusions:**

Increase in quinolone and ceftriaxone NTS is a serious threat to public health requiring continuous surveillance and use of appropriate screening tests for laboratory detection.

## Background

Non typhoidal salmonellae (NTS) are associated in approximately 5% of the patients with bacteremia, invasive infections and death [1]. Increased antimicrobial resistance has made empiric antibiotic of choice for these potentially fatal infections quite limited. Current literature recommends either a fluoroquinolones or a third generation cephalosporins as drug of choice; however increasing evidence of emergence of resistance against these antibiotics in a great concern [2,3].

Outbreaks due to highly resistant NTS have been reported from a number of countries [3-6]. Surveillance of antimicrobial resistance amongst NTS is crucial, particularly in developing countries where facilities for culture and susceptibility testing are not widely accessible and clinicians rely mainly on empirical therapy. While increased resistance in typhoidal Salmonellae has been reported from Pakistan, antimicrobial resistance data of NTS is unavailable. Resistance to quinolone and 3^rd ^generation cephalosporin with extended spectrum beta lactamase (ESBL) producing NTS have not been yet reported from this region. We have assessed antimicrobial susceptibility of NTS from Pakistan over a period of 17 years including analysis of resistance to quinolones and 3^rd ^generation cephalosporin.

## Methods

### Setting

This study was conducted from 1990-2006 at the Aga Khan University (AKU), a tertiary care hospital in Karachi, Pakistan. The hospital and laboratory are accredited with Joint commission of international accreditation (JCIA). Laboratory routinely participates in external quality control surveys with College of American pathologists (CAP). Clinical microbiology laboratory receives specimens across the country via satellite centers in 50 major cities and towns of Pakistan. The data presented here was not collected in a programmed survey but obtained in routine analysis of specimens submitted to the laboratory. All specimens although were processed in laboratory based in Karachi but the data represents strains prevalent across the country. This study was a retrospective analysis of the laboratory data and did not include any identifiable information from the patients therefore formal ethical committee approval was not obtained as per research guidelines of the institute.

### Specimen Selection

Data including both invasive and non-invasive NTS was retrieved from a central computerized database. Duplicate specimens from same patients and samples with *Salmonella enterica *Typhi and Paratyphi were also excluded.

### Microbiological Methods

During the study period NTS from stool and urine were identified biochemically using conventional tests [7]. Isolates from blood, tissues and other sterile body sites were identified using API 20E (Bio Merieux France). Suspected colonies were tested with Salmonella polyvalent antisera (A-I and Vi) (Difco). Serogroups were determined using the slide agglutination method. Non typeable *Salmonella *spp. were those isolates that have biochemical (API 20E) and serological profile suggestive of Salmonella but were not further classified into serogroups due to limited availability of specific O antisera (only A, B, C and D). Serotypes were determined by tube agglutination method against H antigen. If an isolate was not serotyped with the available H antisera only serogroups were reported.

Antimicrobial susceptibility testing was performed as per Clinical Laboratory Standards Institute (CLSI) criteria [8] against nalidixic acid (30 μg) ciprofloxacin (5 μg), ampicillin (10 μg) ceftriaxone (30 μg), chloramphenicol (30 μg), and cotrimoxazole (1.25/23.75 μg). ESBL production was detected by combined disc method (ceftriaxone alone and ceftriaxone-clavulanic acid) [8]. ESBL producing strains were further tested against amoxicillin-clavulanic acid (20/10 μg), gentamicin (10 μg), amikacin (30 μg), piperacillin-tazobactam (100/10 μg) and meropenem (10 μg). Since year 2002 susceptibility against ciprofloxacin was determined by nalidixic acid screening method as recommended by Hakanen et al [9]. Minimum inhibitory concentrations against ciprofloxacin were determined using agar dilution method [8]. Isolates that were resistant to ampicillin, chloramphenicol and cotrimoxazole were considered multidrug resistant (MDR).

### Statistical Analysis

Clinical data were analyzed using SPSS version 15.0 software. Comparisons were made between resistant and sensitive strains in terms of age, gender, specimen source and serogroups. Proportions were compared using the Chi-square test or Fisher's exact test, where appropriate. A p value of less than 5% was considered as statistically significant.

## Results

During the study period, a total of 1967 NTS were identified. A characteristic seasonal pattern in isolation of NTS was observed with increased isolation in the months of May-August corresponding with the summer and monsoon season (data not shown). Majority of NTS isolates were from females (57.5%). Isolation rate of NTS was higher in the age group under 5 years (55.5%). Out of 1967 isolates majority were from stool (91%) followed by blood (6%). Most prevalent organisms were the *Salmonella *group B (659) including *Salmonella enterica *Typhimurium (257) followed by *Salmonella *group C (465), *Salmonella *group A (62) and *Salmonella *group D (48). Due to limited availability of a number of antisera the large group of non-typeable *Salmonella *(728) could not be speciated.

**Figure 1:**
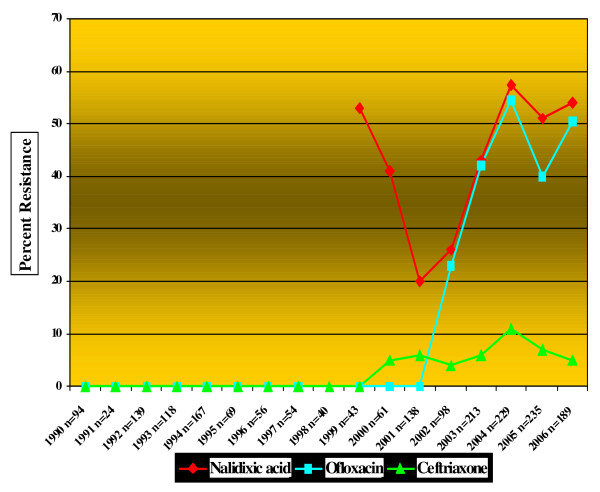
**Showing marked increase in resistance against nalidixic acid, ofloxacin and ceftriaxone in NTS isolates at the Aga Khan University Hospital (1990-2006)**.

**Figure 2:**
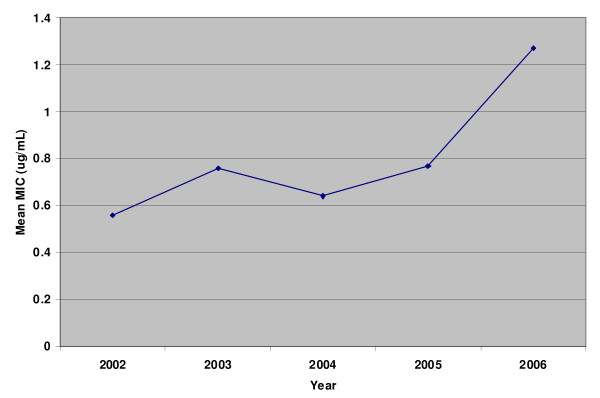
**Showing gradual increase in ciprofloxacin MICs in NTS isolates at the Aga Khan University Hospital (2002-2006)**.

The resistance rates against nalidixic acid, ofloxacin and ceftriaxone were increased (Fig. 1). This increase was significant for nalidixic acid and ofloxacin (p value for trend < 0.001). Between 1990 and 2001 in accordance with NCCLS guidelines interpretative criteria of Enterobacteriaceae was used to report susceptibility of NTS against fluoroquinolone, hence resistance was not detected. However, following the introduction of nalidixic acid screening method in 2002, 27% of the NTS isolates were noted to have reduced susceptibility to quinolones. Mean MIC values of ofloxacin in NTS also gradually increased during this period (Fig 2).

Ceftriaxone resistant NTS first observed in year 2000, increased gradually over the years. ESBL production was seen in 98.7% (n = 78) of ceftriaxone resistant NTS. ESBL producing NTS isolates further showed resistance against amoxicillin clavulanic acid (57%), gentamicin (69%), amikacin (44%) and piperacillin/tazobactam (30%). Resistance to carbapenem was not observed. Ceftriaxone resistance was significantly higher (p value < 0.001) amongst organisms isolated from patients less than 1 year of age, in invasive isolates, in hospitalized patients and in *Salmonella enterica *Typhimurium. In comparison to other *Salmonella *spp., *Salmonella enterica *Typhimurium had significantly higher resistance to ciprofloxacin (35.5 vs. 19%) and had higher MDR rate (39% vs. 8%).

**Figure 3:**
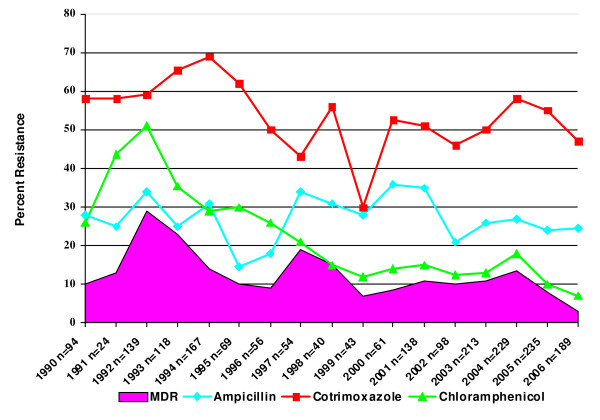
S**howing trends of resistance against first line drugs in NTS isolates at the Aga Khan University Hospital (1990-2006)**.

Interestingly resistance against the first line drugs did not increase over the years. Rather resistance to chloramphenicol decreased from 26% in 1990 to 7% in 2006 (p value < 0.001). Similarly resistance against cotrimoxazole and ampicillin remained static with minimum variations. Isolation of MDR NTS strains significantly decreased to 3% in 2006 (p value < 0.001) (Fig 3).

## Discussion

Globally empirical therapy of invasive NTS infections has become challenging with the emergence of ceftriaxone and quinolone resistance. This is the first report from Pakistan reporting ESBL producing NTS isolates and increase in quinolone. We also observed recent decline in isolation of MDR NTS strains.

Since 1991 ceftriaxone resistant NTS has been reported in various studies [6,10] and is reportedly mainly due to ESBL (particularly CTX-M types) and AmpC β lactamases [3,11]. Within our samples, ceftriaxone resistant NTS were first isolated in the year 2000 and the rate of isolation of these strains has gradually increased over the years. 98.7% of our strains were ESBL producers and showed concurrent resistance to broad spectrum antibiotics including amoxicillin-clavulanic acid, amikacin, gentamicin and piperacillin-tazobactam. Fortunately carbapenem resistance, although reported previously in NTS was not detected in our study [12]. The significant association of these strains with patients under than 1 year of age and with invasive infections makes the management of these strains more problematic. Another concern is inappropriate detection of such resistant strains in most of the laboratories in Pakistan.

This study also reports an increase in fluoroquinolone resistance from 23% in 2002 to 50.5% in 2006. Such an increase is in agreement with reports from other countries [4,5]. Prior to 2002 we were using CLSI breakpoints recommended at the time and were therefore not able to detect any resistance [8]. From 2002 onwards nalidixic acid screening method was employed for the detection of quinolone resistance. The rise in resistance rates correlates also with increase in mean MIC values for these strains. We recommend that accurate detection of *Salmonella *strains with reduced quinolone susceptibility should be reported by the clinical laboratories in Pakistan, as clinical outcome with quinolones therapy is poor.

*Salmonella *Typhimurium had highest resistance rates to ceftriaxone, ciprofloxacin and MDR. Increased resistance to first line drugs has been reported previously in *Salmonella *Typhimurium from UK and Spain [13,14]. Another study from England and Wales has also reported higher ceftriaxone resistance rates in this serotype [10]. Increased antimicrobial resistance in *Salmonella *Typhimurium is alarming as high mortality and invasive infections are associated with this serotype [15,16].

We also observed static resistance rates to ampicillin and cotrimoxazole and a decline in resistance to chloramphenicol and MDR strains as reported previously in Kenya [17]. Another report from Pakistan reporting resistance rates in *Salmonella *Typhi also demonstrated declining resistance to first line drugs [18]. They correlated this with the overall antimicrobial consumption at population level in Karachi and showed a steady reduction in the use of the above antibiotics especially chloramphenicol. Based on their findings we can assume similar correlation in NTS as well.

## Conclusions

Increased resistance to ciprofloxacin and emergence of ESBL producing NTS pose both diagnostic and management dilemma in a developing country like Pakistan. Measures should be taken to stop further emergence of resistance and their dissemination on a priority basis. Preventive strategy would require improved laboratory services, continuous surveillance and restriction of use of broad spectrum antibiotics.

## Competing interests

The authors declare that they have no competing interests.

## Authors' contributions

KJ initiated, planned and completed the study. She also wrote the initial manuscript and SI, AZ, EK and RH critically analyzed it for intellectual content and revised it accordingly. VM performed the data analysis. All authors approved the final version.

## Funding Source

None.

## Pre-publication history

The pre-publication history for this paper can be accessed here:

http://www.biomedcentral.com/1471-2334/10/101/prepub
